# The Central Organisation of the Public Medical Services

**Published:** 1907-05-25

**Authors:** 


### Public Health and Hygiene.

### THE CENTRAL ORGANISATION OF THE PUBLIC MEDICAL SERVICES.

### SHOULD THERE BE A MINISTER OF HEALTH?

In the House of Commons on May 9 Mr. Ramsay Macdonald asked the Minister of Education whether he proposed to create a medical department in connection with the Board of Education to supervise local work done in the medical inspection of school children, and whether legislation would be necessary in order to create such a department. It appears from the answer that legislation will be necessary, and we hope that when the Government take this in hand they will do so in a comprehensive manner and refuse to subordinate considerations of health to those of education.

A recent article in the *Morning Post* states:—

"It is necessary that there should be from the first a medical department at the Board of Education, directly responsible to the Minister for the guidance of the medical officers throughout the country. Otherwise the inspection will be neglected, for it cannot easily be enforced; or, worse still, it will be done in a perfunctory and slipshod manner by medical men ignorant of their duties for local authorities indifferent to the whole matter.

"The duties of a school physician are manifold and call for either special training or special guidance. Nearly 90 per cent. of the issues involved are technical questions of purely educational and pedagogical importance affecting the educational progress of the race. The remaining 10 per cent. are concerned with minor issues, such as the prevention of infectious and contagious disease, and the sanitary condition of the school premises.

"Medical inspection of schools does not concern the Local Government Board, and if the supervision of the inspectors is entrusted to that Board the result in practice will be the mere examination of school drains and of such other trifles as may occur to the medical officer of health. What has the Local Government Board to do with the physiology of educational methods or with the relations of medicine and pedagogics, or with such problems as mental deficiency, defective vision, defective teeth, and 'special schools'? Yet these are the most essential matters with which the school physician will be concerned."

If the Local Government Board has nothing to do with physiology, we may well ask what the Board of Education has to do with medicine? The one board is the central authority of health, the other of education, but neither should be water-tight compartments impervious to the concerns of the other. If the Local Government Board were ostensively and exclusively the central authority of health, as the Board of Education is of education, there would be no question as to its concern in any problem of State medicine, pedagogic or otherwise. Moreover, we disagree entirely with the estimate of our contemporary that nearly 90 per cent. of the issues involved in the duties of the school physician are technical questions of "purely educational importance affecting the educational progress of the race." Where medicine becomes the handmaiden of education, as in such case she would, we admit that the Board of Education is chiefly concerned. Faulty educational methods to be rectified in consultation with the physician are not an unimportant reform and are even urgently required. But these are not the considerations which have quickened the national conscience to a sense of the serious issues involved in the known deplorable physical condition of the children.

No; it is the health aspect of the problem, and not the educational that has stirred the public imagination. The question is so important that educational considerations do not arise until it is settled. It is obviously absurd to provide for the education of the child before you have secured the elementary conditions of mere animal survival. The amazing thing is that for so long it has been thought possible to ignore the primary claims of health in the supposed interests of those of education. And the very men who have insisted most strenuously on the need for rectifying the perspective, the medical officers of health, who by the facts they have elucidated have been chiefly instrumental in bringing the question into the domain of practical politics, it is now urged are unfitted to deal with this health problem, because in their hands it will resolve itself into "a mere examination of school drains and such other trifles as may occur" to them. The medical officer of health no longer is to concern himself "respecting all the influences affecting or threatening to affect injuriously the public health, nor to inquire into the causes, origin, and distribution of diseases within his district," but he is to become a slightly mitigated inspector of nuisances. The medical officer of schools, who, so far as he at present exists is the medical officer of health, is to become a pedagogue faintly tinctured by a medical training, nine-tenths of whose work is to be outside the real work of preventive medicine, and is to be largely devoted to the "physiology of educational methods" and such other bypaths of medicine as, exclusively followed, would constitute a class of quasi-medical practitioners.

But medicine must not be disintegrated into fragments incapable of identification with that historic body of thought and practice of which, as a profession, we are so justly proud. We protest against the creation of a body of medical pedagogues, just as we resent the attempt to degrade the medical officer of health to the level of a mere inspector of drains. We hold that if medicine is to be saved from this fissiparous process it is by the creation of a central department of health presided over by a minister. And we are opposed to the alternative method, that of the appointment of medical officials intent on subordinating medicine to departments of State, which, after all, are of secondary importance to the great interest with which medicine is identified. We are glad to note that the suggestion that a Minister of Health to represent in the Cabinet this great national concern is being canvassed as a public and not as a mere professional interest. Mr. A. Rolland Rainy, M.P., delivered on the 10th inst. an address at the New Reform Club on "The Necessity for a Minister of Public Health," and a resolution in favour of creating such an office was adopted.

The care of health is a matter of such prime importance to the nation, taking precedence in all well-regulated minds of so many other interests which have received priority of political consideration, that makeshift arrangements will no longer satisfy in the dispositions to be made for its further safeguarding. What is needed in the first instance is central organisation, the creation of a department of health, with a Cabinet Minister at its head. To the Minister of Health might be entrusted the re-organisation of the public medical services. This should not definitely be undertaken until the report of the Royal Commission on the Poor-law now sitting has been issued, and until it is, whatever of new work has to be compassed might safely be left to the existing services, for this is certain: the competency of the medical officers in the different services is beyond question. It is in organisation that inadequacy is apparent.

